# Efficacy and Tolerability of Telaprevir for Chronic Hepatitis Virus C Genotype 1 Infection: A Meta-Analysis

**DOI:** 10.1371/journal.pone.0052158

**Published:** 2012-12-20

**Authors:** Yuan Kong, Xiaoping Wang, Yushu Shang, Paul M. Schroder, Wenhua Liang, Xiaoting Ling, Zhiyong Guo, Xiaoshun He

**Affiliations:** 1 Organ Transplant Center, The First Affiliated Hospital, Sun Yat-sen University, Guangzhou, China; 2 Department of Medical Microbiology and Immunology, University of Toledo College of Medicine, Toledo, Ohio, United States of America; Saint Louis University, United States of America

## Abstract

**Background:**

Chronic hepatitis C virus (HCV) infection is one of the leading causes of hepatic cirrhosis and hepatocellular carcinoma, and HCV genotype 1 is the most prevalent genotype and is resistant to current standard therapy. We performed this meta-analysis to evaluate the efficacy and safety of telaprevir-based therapy for chronic HCV genotype 1 infection.

**Methods:**

We included randomized controlled trials with no year or language restriction. All data were analyzed using a random-effects model by Review Manager v5.0. The primary outcome was the proportion of patients achieving sustained virologic response (SVR), and the secondary outcomes were HCV relapse rate, incidence of severe adverse events (SAEs), and discontinuation due to adverse events.

**Results:**

The proportion of achieving SVR was significantly higher in the telaprevir group (odds ratio [OR] = 3.40 [1.92, 6.00], *P*<0.0001; *I^2^* = 87%) regardless of a patients’ previous treatment status. It was also significantly higher in the 24-week and 48-week treatment groups (OR = 4.52 [2.08, 9.81], *P*<0.001; *I^2^* = 85%, and OR = 4.05 [1.56, 10.56], *P* = 0.004; *I^2^* = 92%, respectively), while it was comparable in the 12-week treatment group (OR = 1.32 [0.63, 2.75], *P* = 0.46; *I^2^* = 35%). In addition, the HCV relapse rate was significantly reduced in the telaprevir group (OR = 0.28 [0.16, 0.49], *P*<0.001; *I^2^* = 76%). However, the incidence of SAE (OR = 1.56 [1.15, 2.10], *P* = 0.004; *I^2^* = 0%) and study discontinuation due to adverse events (OR = 2.24 [1.43, 3.50], *P*<0.001; *I^2^* = 37%) were significantly higher in the telaprevir group.

**Conclusion:**

Despite its higher incidence of SAEs and discontinuation due to adverse events, telaprevir-based therapy can increase the proportion of achieving SVR in both previously treated and untreated chronic HCV-1 infected patients.

## Introduction

Hepatitis C virus (HCV) infection is a serious public health concern that affects 170 million people worldwide and has become the leading indication for liver transplantation [1]. Approximately 80% of infected patients fail to clear the virus and less than 10% may develop severe liver diseases such as chronic hepatitis, liver cirrhosis, or hepatocellular carcinoma, which can compromise patient survival [2]. And the number of people requiring treatment for chronic HCV infection is expected to increase over the next 10–20 years [3].

The current standard treatment of chronic hepatitis C recommended by the 2009 American Association for Study of Liver diseases Practice Guidelines [4] is the combined use of the nucleoside analog ribavirin and pegylated interferon alpha, the success of achieving sustained virologic response (SVR) under such treatment depends largely on the viral genotype [5]. Among the 6 known HCV genotypes (1–6), HCV genotype 1 (HCV-1) is the most prevalent circulating strain in Western Europe and North America and its infection is most refractory to the therapy. For patients with HCV-1 infection, less than 50% could achieve SVR with treatment of peginterferon alfa-2a and ribavirin [6], compared to the 80% SVR in genotypes 2 and 3 patients [7]. However, apart from its significant performance in achieving SVR, there is little evidence supporting the effect of such treatment on clinical outcomes. In addition, this drug combination has been associated with severe adverse effects such as fatigue, nausea and depression, thus precluding treatment for many individuals [8]. All the issues above address the need to develop novel anti-HCV drugs with greater anti-virus effect and safety.

Recent studies of new treatment strategies for chronic hepatitis C have been focused on specifically targeted antiviral therapy, novel interferon products and nucleoside analogs, among which the treatment efficacy of direct acting antiviral agents (DAAs) appears to be the most promising. The advanced DAAs for HCV include inhibitors of the viral NS3/4A protease and the viral NS5B RNA-dependent RNA polymerase [9]–[11]. Telaprevir (VX 950, Vertex), as one of the linear NS3/4A protease inhibitors, has been shown in several clinical trials to substantially enhance SVR in chronic HCV-1 infected patients when used in combination with peginterferon and ribavirin, and potentially shorten the treatment period [12]–[16].

Herein, we performed a meta-analysis of the current reported data in clinical trials to gain a meta-analyzed profile of the efficacy and safety of telaprevir-based therapy in chronic HCV-1 infected patients. This study provides important insights capable of better informing clinical physicians regarding the updated treatment strategy for chronic HCV infection.

## Methods

### Patients and Methods

This meta-analysis was carried out according to the Cochrane Reviewer’s Handbook recommended by The Cochrane Collaboration.

### Study Objective

The primary end point was to identify the effects of telaprevir plus peginterferon and ribavirin combination therapy on the proportion of patients who achieved SVR, which was defined as the patients who had undetectable plasma HCV RNA 24 weeks after the last planned dose of the study treatment. The secondary end points were relapse rate, which was defined as a detectable HCV RNA level during the post-treatment follow up period in a patient who had undetectable levels of HCV RNA at the end of the treatment period, and safety, as assessed by the percentage of patients who discontinued therapy due to adverse events and the incidence of severe adverse events (SAE).

### Literature Search

Studies were retrieved using Medline, Embase, Cochrane Database of Systematic Reviews and manual searches with no year or language restrictions. Searching strategies were described as #1: “telaprevir” OR “VX-950” OR “Vertex” OR “NS3/4A protease inhibitor”; #2: “chronic hepatitis C” OR “chronic HCV infection”; or #1 AND #2. General reviews, meta-analyses, and references from published randomized controlled trials (RCTs) and presentation to the International Association for the Study of the Liver, the European Association for the Study of the Liver, and the American Association for the Study of Liver Diseases meetings, were also searched for additional citations.

### Inclusion Criteria

We selected RCTs published as full articles in journals and RCTs published as abstracts in international, European, or American meetings, evaluating telaprevir plus peginterferon and ribavirin combination therapy in chronic hepatitis C genotype 1 patients, with the current standard peginterferon and ribavirin therapy as the control group. Reported results must include the proportion of achieving SVR, relapse rate, incidence of discontinuation and severe adverse events.

### Exclusion Criteria

Nonrandomized trials, trials that included patients with non-type C hepatitis or non-genotype 1 HCV infection, and trials that included patients infected by more than one type of hepatitis virus or HCV genotype but failed to provide the specific outcome data for genotype 1 HCV infected patients were excluded. Trials that did not use the peginterferon and ribavirin combination therapy for 48 weeks as control were excluded as were trials using response-guided therapy. RCTs were also excluded when they didn’t use SVR as an outcome. And trials labeled as being at “high risk of bias” during assessment of bias risks should be excluded from the analyses. In the event of unpublished virologic data, a request for information was made to investigators before excluding the trials from this report.

### Patient Characteristics, Diagnoses, and Treatments

The following items were recorded as potentially useful in assessing clinical heterogeneity between RCTs: study population, method of diagnosing chronic hepatitis C, time from diagnosis, previous treatment and response status to previous treatment, baseline viral load, dose and duration of telaprevir administration, dose and duration of peginterferon and ribavirin administration.

### Selection and Data Extraction

Two reviewers (YK and YS) first screened titles and abstracts, yielding potentially eligible publications. All RCTs considered for inclusion were analyzed independently by the two reviewers. When disagreements regarding bias assessment arose, a third party (ZG) was brought in to resolve the disagreement. The resutls of the individual trials were blinded to the reviewers, and the decision on inclusion or exclusion was not related to the results or conclusions of each manuscript.

### Quality Assessment of Trials Included

A quality assessment was carried out for all the retrieved RCTs. Quality in a systematic review essentially refers to the absence of biases. To assess the methodological validity of the RCTs included in this meta-analysis, we used the Risk of Bias table from Review Manager 5.0 (The Cochrane Collaboration, Oxford, United Kingdom). The following aspects were evaluated: adequate sequence generation, allocation concealment, blinding, incomplete outcome data addressed, free of selective reporting, free of other bias, and intention-to-treat analysis. Trials were excluded from the analyses if they were assessed as at high risk of bias. We used Begg’s funnel plot analysis to test publication bias of the included RCTs. Articles were assessed by two reviewers (YK and YS) independently. Disagreements were resolved by consultation with a third reviewer (ZG).

### Statistical Analysis

All analyses were performed with the intention-to-treat (ITT) method, in which case all randomized patients were included, and patients without the outcome were considered as a treatment failure. The primary outcome was the proportion of patients achieving SVR, and secondary outcomes were HCV relapse rate, incidence of SAEs, and discontinuation due to adverse events. A primary analysis including all of the patients and subgroup analyses regarding treatment duration and previous treatment status were planned. We used the odds ratio as a measurement of effect size for SVR, relapse, drug discontinuation, and SAE. The DerSimonian and Laird model for random-effects meta-analysis was adopted to obtain summary estimates across the included studies. We tested for heterogeneity using the Cochran Q test, which follows a chi-square distribution, to make inferences about the null hypothesis of homogeneity. The *I^2^* value was used to demonstrate the percentage of inter-study variation in all variation (including inter-study variation and system error). If *I^2^*>50%, subgroup analysis, meta-regression analysis and sensitivity analysis would be applied if possible. All statistical analyses were performed using the statistical software Review Manager 5.0 (The Cochrane Collaboration, Oxford, United Kingdom). This report follows the PRISMA guidelines [13] and the Cochrane collaboration guideline for reporting meta-analyses.

## Results

### Characteristics of Included Studies

**Figure 1:**
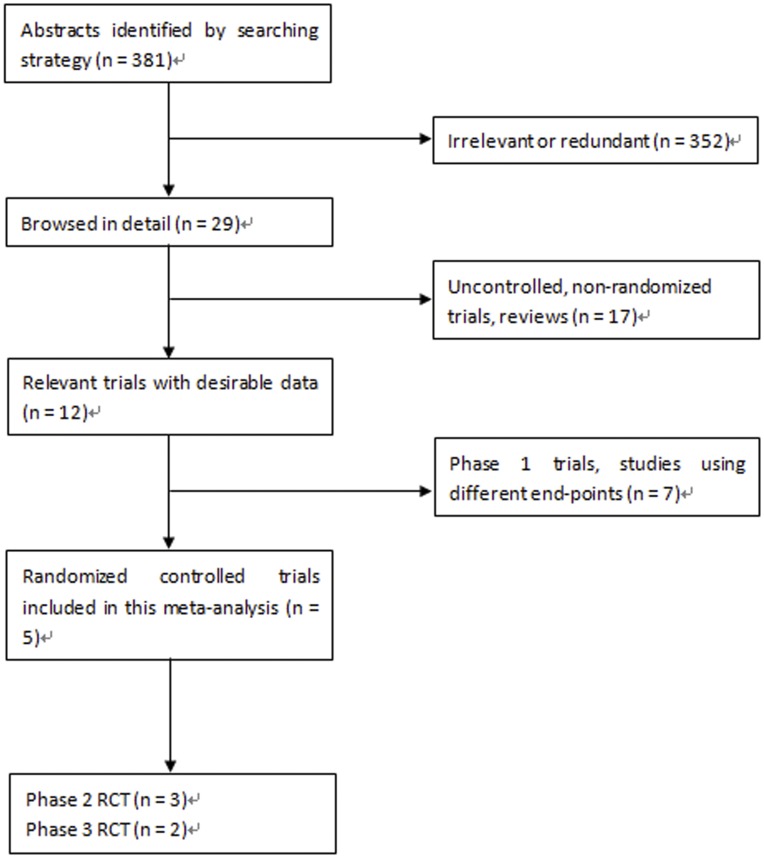
Flow diagram of study identification.

Figure 1 shows the process of study selection. 381 citations were initially found and we included 5 RCTs with 2776 patients in this meta-analysis, including 3 phase 2 clinical trials [12]–[14] with 1026 patients and 2 phase 3 trials [15], [16] with 1750 patients. The patients in all 5 RCTs were 18 to 70 years of age and had HCV-1 infection with evidence of chronic hepatitis, as confirmed by a liver biopsy within 18 months before enrollment. The 5 RCTs were conducted in both previously untreated and treated patients. Telaprevir was given as a single dose of 1250 or 1125 mg on study day 1 and followed by a dose of 750 mg every 8 hours orally in the 3 phase 2 trials, and administered orally at a dose of 750 mg every 8 hours with food in the 2 phase 3 trials. In all 5 RCTs, peginterferon alfa-2a was administered by subcutaneous injection at a dose of 180 ug per week, and ribavirin orally at a dose of 1000 mg per day (in patients who weighed less than 75 kg) or 1200 mg per day (in patients who weighed 75 kg or more). The duration of telaprevir treatment was 12 weeks in combination with 12, 24, or 48 weeks of peginterferon and ribavirin, while the control treatment of peginterferon and ribavirin lasted 48 weeks. Table 1 summarizes the characteristics of the included studies.

**Table 1:** Characteristics of included studies.

Studies	Number of Patients (Telaprevir/PR)	Gender (male/female)	White Popula-tion	Geno-type	Previous Treatment Status	Study Design	Telaprevir Dose
Hezode 2009^13^	241/82	192/131	304 (94%)	1	untreated	T12PR24/T12PR48/T12P12/PR48	1250 mg on day 1; followed by 750 mg Q8H
McHutchison 2010^14^	339/114	306/147	402 (89%)	1	treated	T12PR24/T24PR48/T24PR24/PR48	1125 mg on day 1; followed by 750 mg Q8H
McHutchison 2009^15^	175/75	157/93	192 (77%)	1	untreated	T12PR12/T12PR24/T12PR48/PR48	1250 mg on day 1; followed by 750 mg Q8H
Jacobson 2011^16^	727/361	636/452	958 (88%)	1	untreated	T12PR24/T12PR48/T8PR24/T8PR48/PR48	750 mg Q8H
Zeuzem 2011^17^	530/132	460/202	615 (93%)	1	treated	T12PR48/PR48	750 mg Q8H

T, telaprevir; P, peg-interferon; R, ribavirin; Q8H, every 8 hours.

### Bias Risks

**Figure 2:**
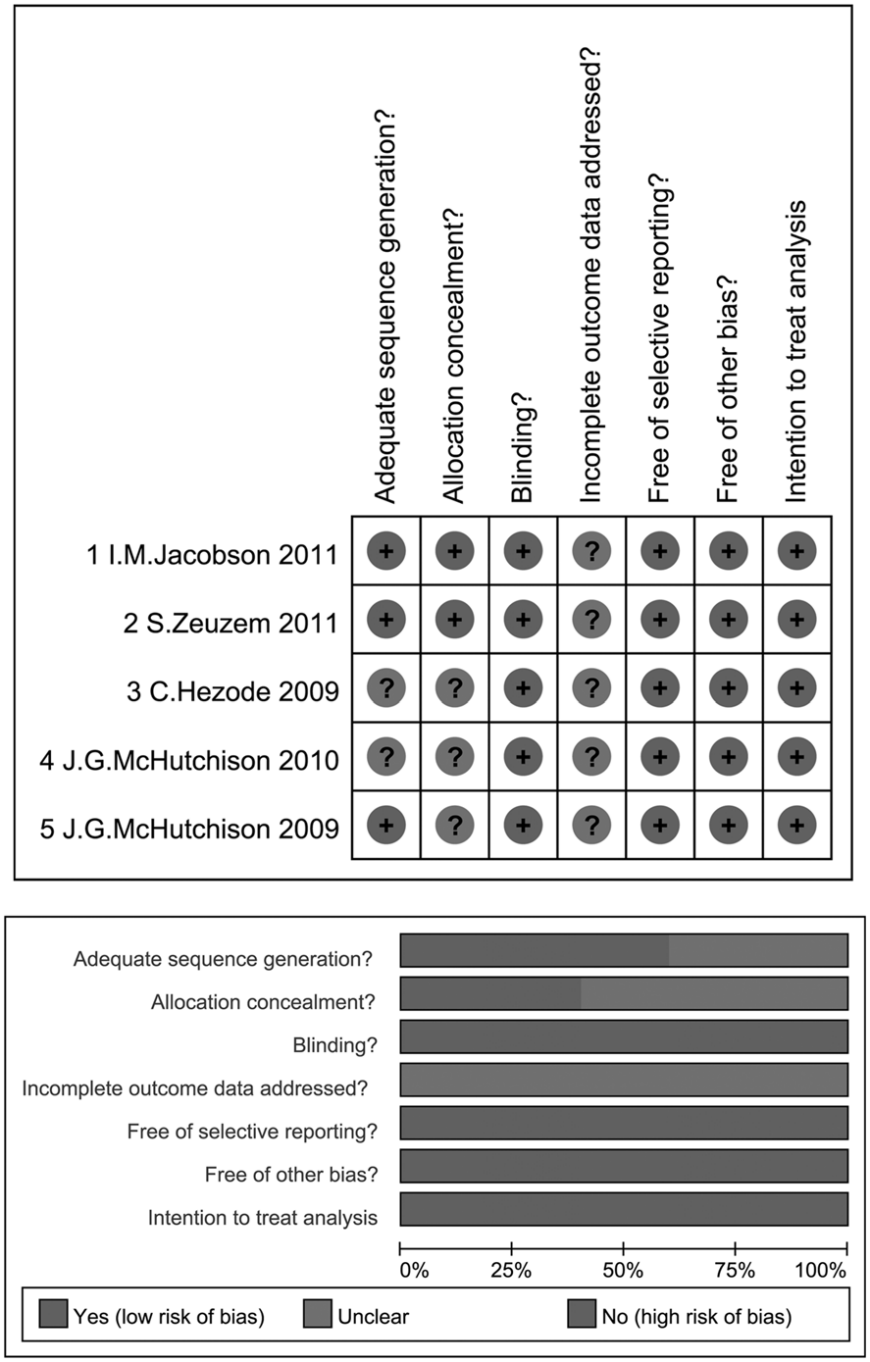
Risk of bias of the included studies. All 5 RCTs adopted blinding and intention-to-treat analysis and were free of selective reporting or other bias. Adequate sequence was generated in 3 and allocation concealed in 2 of the 5 RCTs. Whether and how the incomplete outcome data were addressed was unclear in all 5 RCTs. Each row represents each study and the column represents the risk of bias to be evaluated.

To address the strength of evidence in this meta-analysis, we evaluated the risk of bias of the 5 RCTs. In general, the included trials were at low risk of bias for most of the aspects evaluated. All 5 RCTs adopted blinding and intention-to-treat analysis and were free of selective reporting or other bias. Adequate sequence was generated in 3 and allocation concealed in 2 of the 5 RCTs. Whether and how the incomplete outcome data were addressed was unclear in all 5 RCTs. Figure 2 summarizes the evaluation of risk of bias. We also tested the 5 included RCTs for publication bias in each comparison of the primary and secondary outcomes. No publication bias was identified in comparisons of SVR (*P* = 0.835), relapse rate (*P* = 0.615), SAE incidence (*P* = 0.387), or discontinuation rate (*P* = 0.295) (Figure S1, S2, S3, S4).

### Effects of Telaprevir Plus Peginterferon and Ribavirin Therapy on SVR

**Figure 3:**
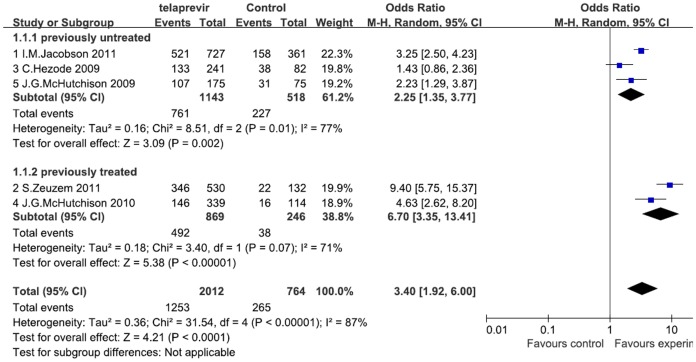
Meta-analysis of telaprevir plus peginterferon and ribavirin therapy on SVR according to previous treatment. The proportion of achieving SVR was significantly higher in the telaprevir group than in the PR group in the previously untreated (OR = 2.25 [1.35, 3.77], *P* = 0.002; *I^2^* = 77%), treated (OR = 6.7 [3.35, 13.41], *P*<0.001; *I^2^* = 71%), and overall population (OR = 3.40 [1.92, 6.00], *P*<0.001; I^2^ = 87%). OR = odds ratio, *I^2^* = heterogeneity index. Columns in green represent the mean difference of each study and column size represents the study weight in the analysis. Lanes represent the 95% CI of each study. Diamonds in black represent the overall effect size, and diamond width represents the overall 95% CI.

We conducted a meta-analysis regarding SVR in all included patients receiving telaprevir-based therapy compared with patients receiving standard peginterferon and ribavirin (PR) therapy (Figure 3). The pooled estimates showed that the overall proportion of achieving SVR was significantly higher in the telaprevir group than in the PR group (OR = 3.40 [1.92, 6.00], *P*<0.001; *I^2^* = 87%).

### Effects of Telaprevir Plus Peginterferon and Ribavirin Therapy on SVR According to Previous Treatment

Three of the 5 RCTs compared the effect of telaprevir in combination with peginterferon and ribavirin on SVR in previously untreated patients [12], [14], [15], and 2 in previously treated patients [13], [16]. We conducted a meta-analysis regarding SVR in patients receiving telaprevir-based therapy compared with patients receiving standard peginterferon and ribavirin (PR) therapy in subgroups defined by previous treatment status (Figure 3). In the previously untreated subgroup, the proportion of achieving SVR was significantly higher in telaprevir group than PR group (OR = 2.25 [1.35, 3.77], *P* = 0.002; *I^2^* = 77%). In the previously treated subgroup, it was also significantly higher than the PR group (OR = 6.70 [3.35, 13.41], *P*<0.001; *I^2^* = 71%).

### Effects of Telaprevir Plus Peginterferon and Ribavirin Therapy on SVR According to Treatment Duration

**Figure 4:**
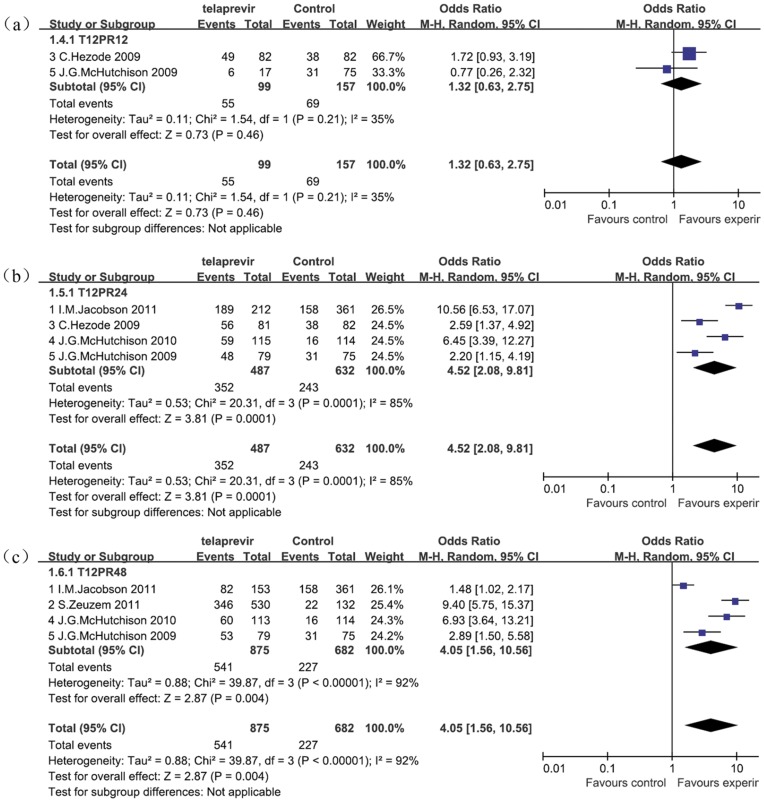
Meta-analysis of telaprevir plus peginterferon and ribavirin therapy on SVR according to treatment duration. There was no statistically significant difference in the proportion of patients who achieved SVR between the T12PR12 subgroup and the PR group (OR = 1.32 [0.63, 2.75], *P* = 0.46; *I^2^* = 35%), while it was significantly higher in the T12PR24 subgroup (OR = 4.52 [2.08, 9.81], *P*<0.001; *I^2^* = 85%) and in the T12PR48 subgroup (OR = 4.05 [1.56, 10.56], *P* = 0.004; *I^2^* = 92%). OR = odds ratio, *I^2^* = heterogeneity index. Columns in green represent the mean difference of each study and column size represents the study weight in the analysis. Lanes represent the 95% CI of each study. Diamonds in black represent the overall effect size and diamond width represents the overall 95% CI.

Two of the 5 RCTs evaluated the 12-week telaprevir in combination with 12-week PR therapy (T12PR12), 4 evaluated the 12-week telaprevir in combination with 24-week PR therapy (T12PR24), and 4 evaluated the 12-week telaprevir in combination with 48-week PR therapy (T12PR48), in comparison with the standard 48-week PR therapy. We conducted a meta-analysis regarding SVR in patients receiving telaprevir-based therapy compared with patients receiving standard PR therapy in subgroups of treatment duration (Figure 4). There was no statistically significant difference in the proportion of achieving SVR between the T12PR12 subgroup and the PR groups (OR = 1.32 [0.63, 2.75], *P* = 0.46; *I^2^* = 35%). In the T12PR24 subgroup, the proportion of achieving SVR was significantly higher in the telaprevir group than in the PR group (OR = 4.52 [2.08, 9.81], *P*<0.001; *I^2^* = 85%). In the T12PR48 subgroup, it was also significantly higher than the PR group (OR = 4.05 [1.56, 10.56], *P*<0.001; *I^2^* = 92%).

### Effects of Telaprevir Plus Peginterferon and Ribavirin Therapy on HCV Relapse Rate

**Figure 5:**
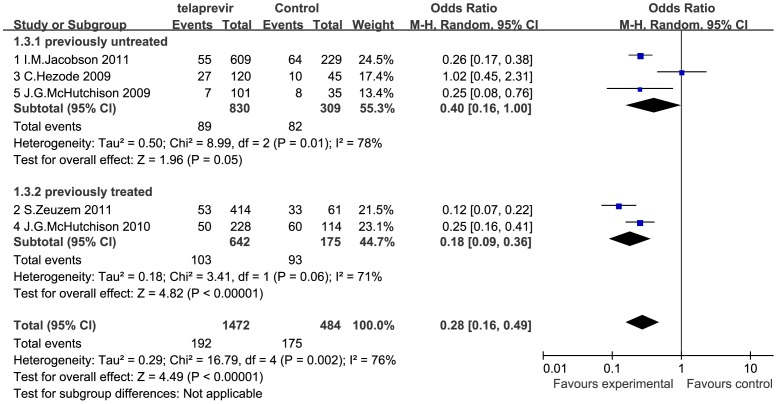
Meta-analysis of telaprevir plus peginterferon and ribavirin therapy on HCV relapse rate according to previous treatment. The incidence of relapse was significantly reduced in previously untreated (OR = 0.40 [0.16, 1.00], *P* = 0.05; *I^2^* = 78%), treated (OR = 0.18 [0.09, 0.36], *P*<0.001; *I^2^* = 71%), and overall population (OR = 0.28 [0.16, 0.49], *P*<0.001; *I^2^* = 76%). OR = odds ratio, *I^2^* = heterogeneity index. Columns in green represent the mean difference of each study and column size represents the study weight in the analysis. Lanes represent the 95% CI of each study. Diamonds in black represent the overall effect size and diamond width represents the overall 95% CI.

Three of the 5 RCTs evaluated the effects of telaprevir plus peginterferon and ribavirin on relapse rate in previously untreated patients [12], [14], [15], and 2 in previously treated patients [13], [16]. We conducted a meta-analysis regarding relapse rate in patients receiving telaprevir-based therapy versus standard PR therapy according to the previous treatment status (Figure 5). In general, relapse was defined as a detectable HCV RNA level during the post-treatment follow up period in a patient who had undetectable levels of HCV RNA at the end of the treatment period. In the previously untreated subgroup, the incidence of relapse was significantly lower in the telaprevir group than in the PR group (OR = 0.40 [0.16, 1.00], *P* = 0.05; *I^2^* = 78%). In the previously treated subgroup, the incidence of relapse was also significantly lower in the telaprevir group than in the PR group (OR = 0.18 [0.09, 0.36], *P*<0.001; *I^2^* = 71%). The pooled estimates showed that the overall incidence of relapse in the previously untreated and treated patients was significantly reduced in the telaprevir group (OR = 0.28 [0.16, 0.49], *P*<0.001; *I^2^* = 76%).

### Safety of Telaprevir Plus Peginterferon and Ribavirin Therapy

**Figure 6:**
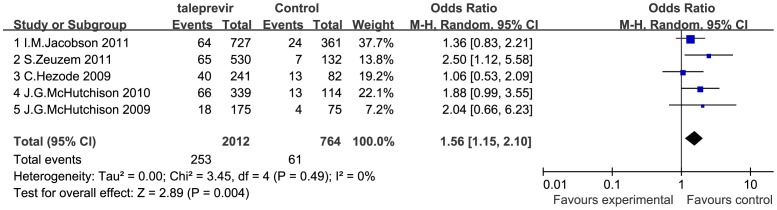
Meta-analysis of telaprevir plus peginterferon and ribavirin therapy on SAE incidence. The incidence of SAE was significantly higher in the telaprevir group than in the PR group (OR = 1.56 [1.15, 2.10], *P* = 0.004; *I^2^* = 0%). OR = odds ratio, *I^2^* = heterogeneity index. Columns in green represent the mean difference of each study and column size represents the study weight in the analysis. Lanes represent the 95% CI of each study. Diamonds in black represent the overall effect size and diamond width represents the overall 95% CI.

**Figure 7:**
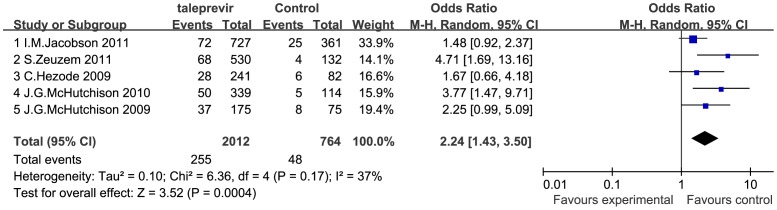
Meta-analysis of telaprevir plus peginterferon and ribavirin therapy on discontinuation incidence. The incidence of therapy discontinuation due to adverse events was significantly higher in the telaprevir group than in the PR group (OR = 2.24 [1.43, 3.50], *P*<0.001; *I^2^* = 37%). OR = odds ratio, *I^2^* = heterogeneity index. Columns in green represent the mean difference of each study and column size represents the study weight in the analysis. Lanes represent the 95% CI of each study. Diamonds in black represent the overall effect size and diamond width represents the overall 95% CI.

Information regarding the incidence of adverse events were sought in all 5 included RCTs, including but not limited to rash, anemia, pruritus, fatigue, flu-like syndrome, headache, nausea, insomnia, diarrhea, and pyrexia, among which rash and anemia are the most common adverse events that occur when telaprevir is added, which can lead to discontinuation of treatment in serious cases. To assess the safety profile of telaprevir-based therapy, we conducted meta-analyses regarding the incidence of SAE and therapy discontinuation due to adverse events in patients receiving telaprevir-based versus standard PR therapy. The incidence of SAE was significantly higher in the telaprevir group than in the PR group (OR = 1.56 [1.15, 2.10], *P*<0.001; *I^2^* = 0%) (Figure 6). The incidence of therapy discontinuation due to adverse events was significantly higher in the telaprevir group than in the PR group (OR = 2.24 [1.43, 3.50], *P*<0.001; *I^2^* = 37%) (Figure 7).

Additionally, meta-analyses regarding the most commonly recognized adverse events, namely rash and anemia, were carried out in patients receiving telaprevir-based versus standard PR therapy. The incidence of rash was significantly higher in the telaprevir group than in the PR group (OR = 2.11 [1.42, 3.14], *P*<0.001; *I^2^* = 68%) (Figure S5). Also, the incidence of anemia was significantly higher in the telaprevir group than in the PR group (OR = 1.88 [1.19, 2.96], *P* = 0.007; *I^2^* = 64%) (Figure S6).

### Heterogeneity

Most of the heterogeneity tests for our analyses showed *I^2^*>50%, indicating significant inter-study heterogeneity. Subgroup analyses were carried out regarding the possible factors that could cause inter-study variation, which also ended in significant heterogeneity. Ideally, a meta-regression analysis should be conducted to address this matter, but we failed to do so since the data on all the potential causes of heterogeneity were incomplete. Sensitivity analysis was carried out. Adjustment of inclusion or exclusion of individual trials or alteration of statistical models came out with the same results, suggesting that the results of these analyses were stable and reliable.

### Trial Sequential Analysis

We also conducted trial sequential analysis due to the small number of included studies and the underlying liability of type I error (Trial Sequential Analysis, Copenhagen Trial Unit, 2011). We calculated the information size required to demonstrate of reject the telaprevir-based effect of a 20% odds ratio reduction, which was 1333 patients. Information size were derived to ensure a maximum type I error of 5%. And it was heterogeneity adjusted, using the estimate of diversity D2. Information size was derived assuming and event proportion of 32% in the control group (median event proportion in this control group). The cumulative Z-curve crossed both the trial sequential monitoring boundaries and the conventional boundaries and surpassed the required information size, thereby confirming that telaprevir-based therapy is superior to PR therapy in increasing the patient proportion of achieving SVR (Figure S7).

## Discussion

This meta-analysis assessed the efficacy and safety of telaprevir-based therapy and 4 major conclusions can be drawn: (1) Telaprevir-based therapy can increase the proportion of achieving SVR in HCV-1 infected patients and reduce relapse rate in comparison with PR therapy; (2) Telaprevir-based therapy can increase the proportion of achieving SVR in both previously treated and untreated patients; (3) Use of telaprevir may shorten the duration of peginterferon plus ribavirin treatment; (4) Telaprevir-based therapy is accompanied with higher incidences of SAEs and treatment discontinuation due to adverse events.

Three of the included RCTs investigated the efficacy and safety of telaprevir in previously untreated patients, and 2 in previously treated patients who were partial responders, non-responders, or relapse patients. While the difference in previous treatment status may increase the risk of heterogeneity and become a potential liability, the subgroup analysis we conducted accordingly demonstrated a boost in achieving SVR in both previously treated and untreated patient subgroup, with the OR value in previously treated subgroup higher than untreated subgroup (6.70 and 2.25, respectively). Such result indicates that HCV-1 is more susceptible to telaprevir-based therapy probably because its inhibition of NS3/4A protease is directly related to HCV replication and assembly [9]. For treatment-naïve patients, telaprevir-based therapy can be the first choice of clinical treatment since it offers a better anti-virus effect. And for previously treated patients, considering HCV-1 drug resistance is the main cause of partial response and no response, the more significant improvement in SVR presented in these patients suggests that telaprevir plus peginterferon and ribavirin therapy can somehow protect the patients from the generation of mutation in targeted genes and thus are less prone to drug-resistance. And between the options of retreatment with the same medications or telaprevir-based regimen in patients who failed the standard PR therapy, the latter may have a better chance of clearing the virus. For both previously treated and untreated patients, the use of telaprevir in combination with peginterferon and ribavirin results in a greater chance of achieving SVR. Although there hasn’t been any prospective blinded study to translate SVR into clinical outcomes, several long-term follow-up studies have shown that the attainment of SVR is durable and predictive of long-term disease remission and clinical benefits in terms of histological improvement and prevention of complications [17], [18]. In fact, AASLD has recently updated its guideline in 2011, recommending to use telaprevir or boceprevir in combination with peginterferon and ribavirin for the treatment of genotype-1 chronic HCV infection in both treatment-naïve patients and patients who have previously received therapy. The results of the current study further support this recommendation.

To address the issue of proper duration and protocol of telaprevir-based therapy, 2 of the RCTs investigated the efficacy and safety of T12PR12 treatment [12], [14], 4 investigated T12PR24 [12], [15] and T12PR48 treatment [13], [16], respectively. Patients assigned to T12PR48 therapy demonstrated a significantly higher SVR proportion compared to the standard PR 48 therapy, which indicates that the improvement in achieving SVR and anti-virus effect can be attributed to the addition of telaprevir. Furthermore, by comparing the T12PR12 and T12PR24 protocols to the PR48 therapy, we assessed whether the addition of telaprevir could shorten the duration of therapy with peginterferon and ribavirin, which was known to cause numerous adverse events [6]–[8]. Patients randomly assigned to the T12PR12 protocol showed no difference in SVR proportion compared with those receiving the 48-week PR therapy in our meta-analyses, which can also be interpreted as no additional anti-virus effect gained by 12-week telaprevir therapy. On the other hand, patients assigned to the T12PR24 protocol demonstrated clear improvement in achieving SVR compared to the 48-week PR therapy. These results support that the addition of telaprevir to the standard PR therapy has a better anti-virus effect, and the equal or even higher SVR proportion achieved by adopting the T12PR12 or T12PR24 therapy can spare the use of peginterferon and ribavirin for 24 or 36 weeks and shorten the total treatment duration.

Another issue that has been brought into sight concerning treatment duration is the application of response-guided therapy in telaprevir protocols. In another open-label study designed to investigate the effects of response-guided therapy, 150 patients with extended rapid viral response (eRVR) to telaprevir and ribavirin treatment were randomized to 24 or 48 weeks of ribavirin to evaluate whether 48-week therapy can gain further benefits [19]. The results showed no further benefit for the 12-week telaprevir plus 48-week ribavirin treatment in patients with eRVR. Collectively, the T12PR24 protocol could be more effective and shorten the therapy duration compared with the conventional PR48 protocol, thus possibly serving as the treatment of choice for chronic HCV-1 infection. However, so far there is no study that has properly addressed this issue. Therefore, whether the T12PR12 therapy has equal or greater benefits compared to the T12PR24 therapy in patients with RVR is remained to be tested.

Patients with hepatitis C are particularly prone to development of drug-resistance upon exposure to protease inhibitors and other HCV antivirals such as ribavirin [20], [21]. The occurrence of drug-resistance to telaprevir is highly related to the patient’s previous treatment status, indicating that HCV genetic changes are more likely to happen in previously treated patients, while in treatment-naïve patients telaprevir resistance due to the presence of HCV variants is uncommon [22]. Nevertheless, this meta-analysis suggested otherwise by showing that telaprevir-based therapy resulted in a higher proportion of achieving SVR and a lower relapse rate in both previously untreated and treated patients, with a higher OR value in the previously treated group in SVR proportion (6.70 versus 2.25) and a lower OR value in relapse rate (0.18 versus 0.40), indicating that the advantage of telaprevir-based therapy over conventional PR therapy is more significant in previously treated patients than in untreated patients and the better virologic response may result from a smaller chance of drug-resistance. However, this is not conclusive since we could not compare the previously untreated group with the treated group directly and the relation between SVR and drug-resistance could not be built in this meta-analysis. Other factors associated with resistance profiles of telaprevir are HCV-1 subtype [23], [24], drug regimen [25], [26] and dosing [27]. Collectively, careful evaluation of the risks of drug resistance, use of telaprevir thrice daily in combination with peginterferon and ribavirin, may prevent patients from resistance to telaprevir.

Safety is the biggest concern when telaprevir is used. Despite its encouraging efficacy in terms of enhanced SVR and decreased relapse rate, telaprevir combination treatment is coupled with an increased incidence of adverse events, including but not limited to rash, anemia, pruritus, anemia, fatigue, flu-like syndrome, headache, nausea, insomnia, diarrhea, and pyrexia, among which rash and anemia are the most common adverse events. These adverse events can lead to discontinuation of treatment in severe cases. The incidences of both SAE and discontinuation of treatment are increased with telaprevir combination treatment, as shown in this meta-analysis. Therefore, the use of telaprevir is limited to interferon-intolerant patients, and caution should be taken when telaprevir is used over a long period of time. Since the currently used PR therapy has already been recognized to have numerous adverse effects, we tried to discover whether the potentially shortened treatment duration by adding telaprevir can reduce the drug toxicity of peginterferon and ribavirin. However, since we failed to compare the incidence of adverse events separately in different PR duration subgroups due to lack of information, we cannot provide solid evidence on this matter and further studies focusing on how to minimize the adverse effects of telaprevir-based therapy should be done. Poor patient compliance should be noted since adherence to medications administrated thrice daily is only 59%, which requires strong and repeated encouragement from all health care providers, and a pillbox should be provided to improve patient compliance [28].

In general, this meta-analysis included all relevant RCTs from various countries and centers, probably providing the best evidence concerning telaprevir treatment for Hepatitis C. However, undoubtedly, there are several limitations in this meta-analysis. First, the primary outcome assessed here is SVR, which is a valid outcome for anti-virus effect and is associated with long-term clinical benefit [17], [18], [29]–[31]. However, with no actual clinical outcomes such as liver enzyme levels, survival, and complications being assessed, whether the achievement of SVR can be directly interpreted as clinical beneficial is still controversial. Second, there are only 5 RCTs included in this meta-analysis and all 5 studies were supported by pharmaceutical industry, which may weaken the strength of evidence demonstrated in this article, although the number of patients included in these 5 RCTs was fairly large and the risk of bias was relatively low. Third, the heterogeneity in most of these analyses is relatively high, probably due to the small number of included RCTs, different study populations and treatment durations, which indicates inter-study variation and may compromise the reliability of these results, although sensitivity analysis showed that the results were stable and reliable. Finally, most of the population studied here are whites, thus the conclusion may not be true in other races and areas. All these limitations point out the direction for future studies.

In conclusion, it is convincing that telaprevir is a highly potent novel drug for chronic HCV-1 hepatitis. However, cautions should be taken with telaprevir combination therapy due to increased risk of adverse events. Future studies should focus on long-term clinical outcomes of telaprevir-based therapy, applying response-guided therapy in telaprevir regimens, minimizing the adverse effects of telaprevir-based therapy, and developing second generation NS3/N4A inhibitors with less adverse effects.

## Supporting Information

Figure S1 **Begg’s funnel plot of publication bias for the SVR comparison with pseudo 95% confidence limits.** Horizontal line represents the overall effect size of the 5 RCTs. Spots represent each RCT. *P* = 0.835. No publication bias was identified.
(TIF)

Figure S2 **Begg’s funnel plot of publication bias for the relapse rate comparison with pseudo 95% confidence limits.** Horizontal line represents the overall OR of the 5 RCTs. Spots represent each RCT. *P* = 0.615. No publication bias was identified.
(TIF)

Figure S3 **Begg’s funnel plot of publication bias for the comparison of SAE incidence with pseudo 95% confidence limits.** Horizontal line represents the overall OR of the 5 RCTs. Spots represent each RCT. *P* = 0.387. No publication bias was identified.
(TIF)

Figure S4 **Begg’s funnel plot of publication bias for the discontinuation rate comparison with pseudo 95% confidence limits.** Horizontal line represents the overall OR of the 5 RCTs. Spots represent each RCT. *P* = 0.295. No publication bias was identified.
(TIF)

Figure S5 **Meta-analysis of telaprevir plus peginterferon and ribavirin therapy on rash incidence.** The incidence of rash was significantly higher in telaprevir group than in the PR group (OR = 2.11 [1.42, 3.14], *P*<0.001; *I^2^* = 68%). Columns in green represent the mean difference of each study and column size represents the study weight in the analysis. Lanes represent the 95% CI of each study. Diamonds in black represent the overall effect size, and diamond width represents the overall 95% CI.
(TIF)

Figure S6 **Meta-analysis of telaprevir plus peginterferon and ribavirin therapy on anemia incidence.** The incidence of anemia was significantly higher in the telaprevir group than in the PR group (OR = 1.88 [1.19, 2.96], *P* = 0.007; I^2^ = 64%). Columns in green represent the mean difference of each study and column size represents the study weight in the analysis. Lanes represent the 95% CI of each study. Diamonds in black represent the overall effect size, and diamond width represents the overall 95% CI.
(TIF)

Figure S7 **The heterogeneity-adjusted required information size to demonstrate or reject a 20% odds ratio reduction of telaprevir-based therapy (with a control group proportion of 32%, an alpha of 5%) is 1333 patients (vertical red line).** The rede inward-sloping line to the left make up the trial sequential monitoring boundaries and the blue line is the cumulative Z-curve.
(TIF)

Table S1 **PRISMA checklist.**
(DOC)
