# Supplementary figures and images for: Efficacy and Tolerability of Telaprevir for Chronic Hepatitis Virus C Genotype 1 Infection: A Meta-Analysis
Source: PLoS One. 2012 Dec 20;7(12):e52158. doi: 10.1371/journal.pone.0052158 (PMC3527389; doi:10.1371/journal.pone.0052158)

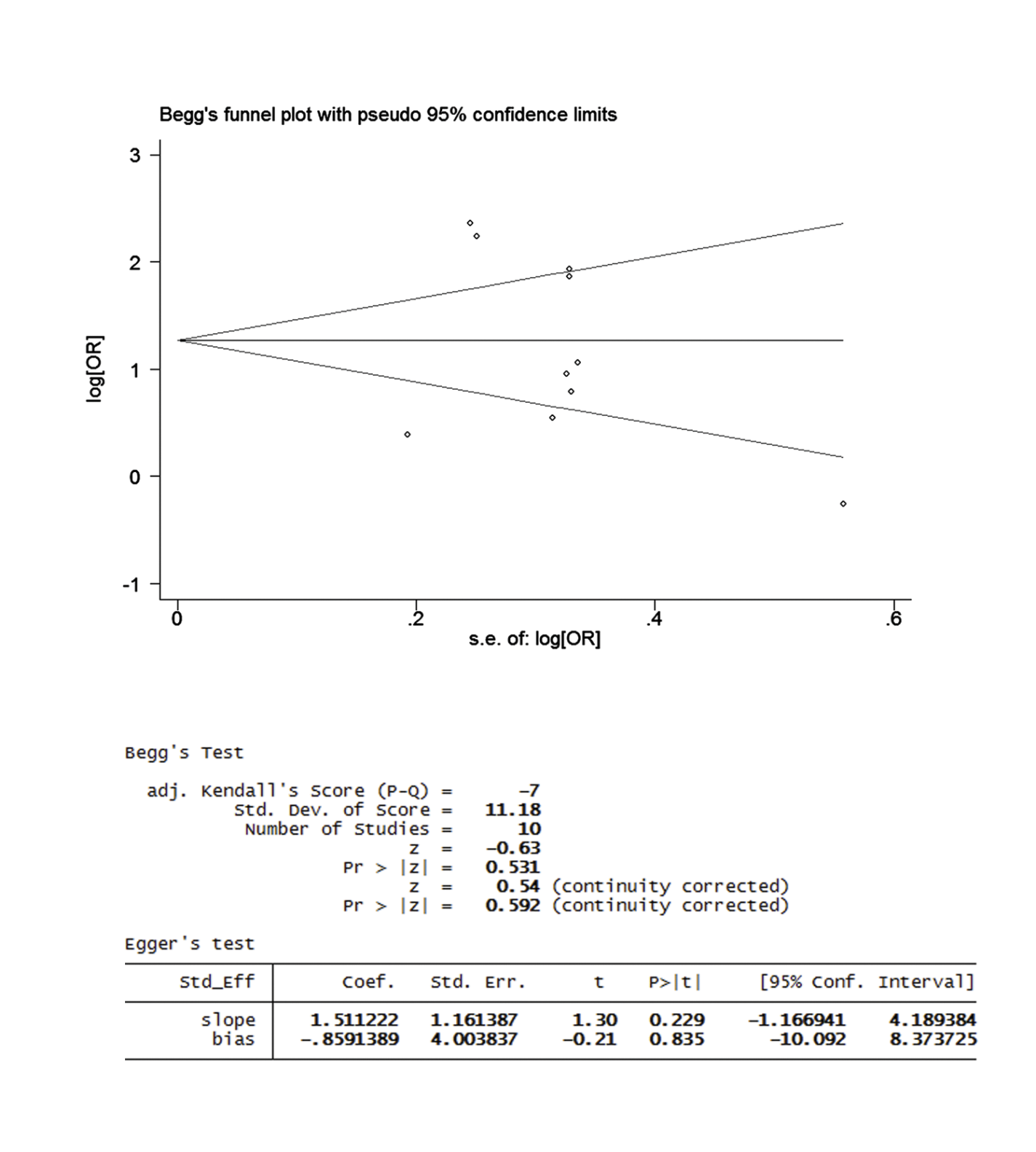

Supplement: Figure S1 — Begg’s funnel plot of publication bias for the SVR comparison with pseudo 95% confidence limits. Horizontal line represents the overall effect size of the 5 RCTs. Spots represent each RCT. P = 0.835. No publication bias was identified. (TIF) [file pone.0052158.s001.tif]

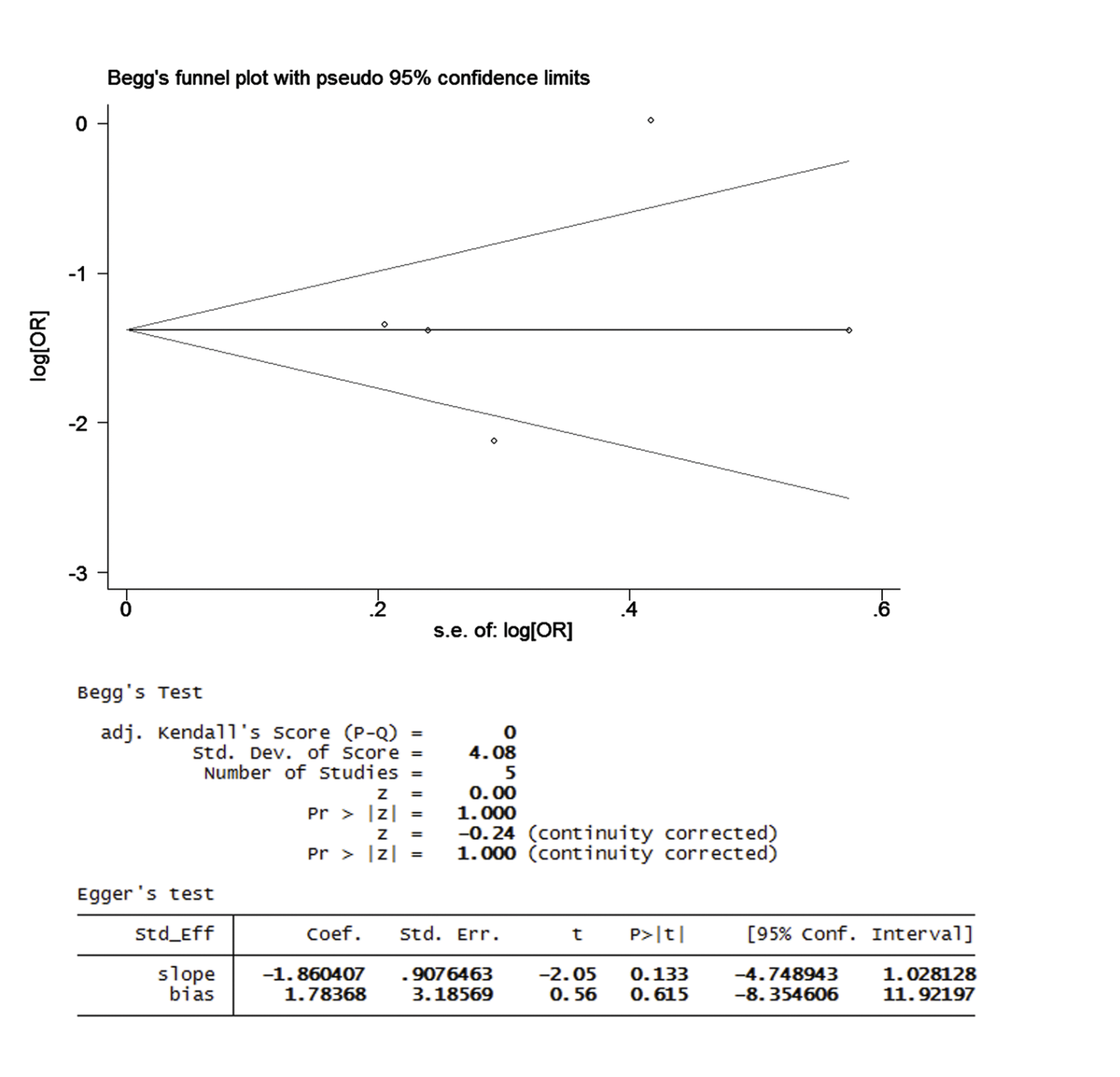

Supplement: Figure S2 — Begg’s funnel plot of publication bias for the relapse rate comparison with pseudo 95% confidence limits. Horizontal line represents the overall OR of the 5 RCTs. Spots represent each RCT. P = 0.615. No publication bias was identified. (TIF) [file pone.0052158.s002.tif]

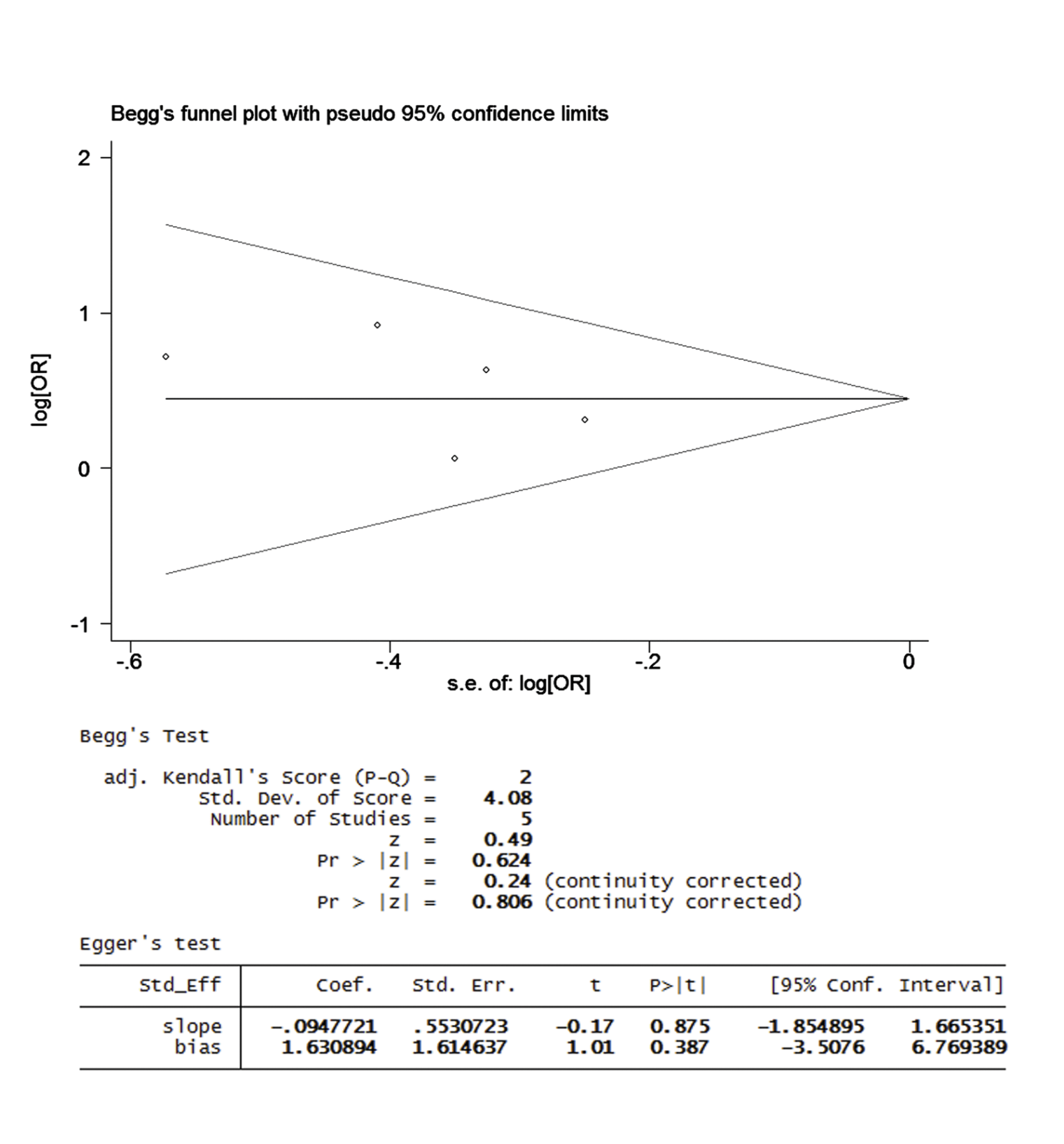

Supplement: Figure S3 — Begg’s funnel plot of publication bias for the comparison of SAE incidence with pseudo 95% confidence limits. Horizontal line represents the overall OR of the 5 RCTs. Spots represent each RCT. P = 0.387. No publication bias was identified. (TIF) [file pone.0052158.s003.tif]

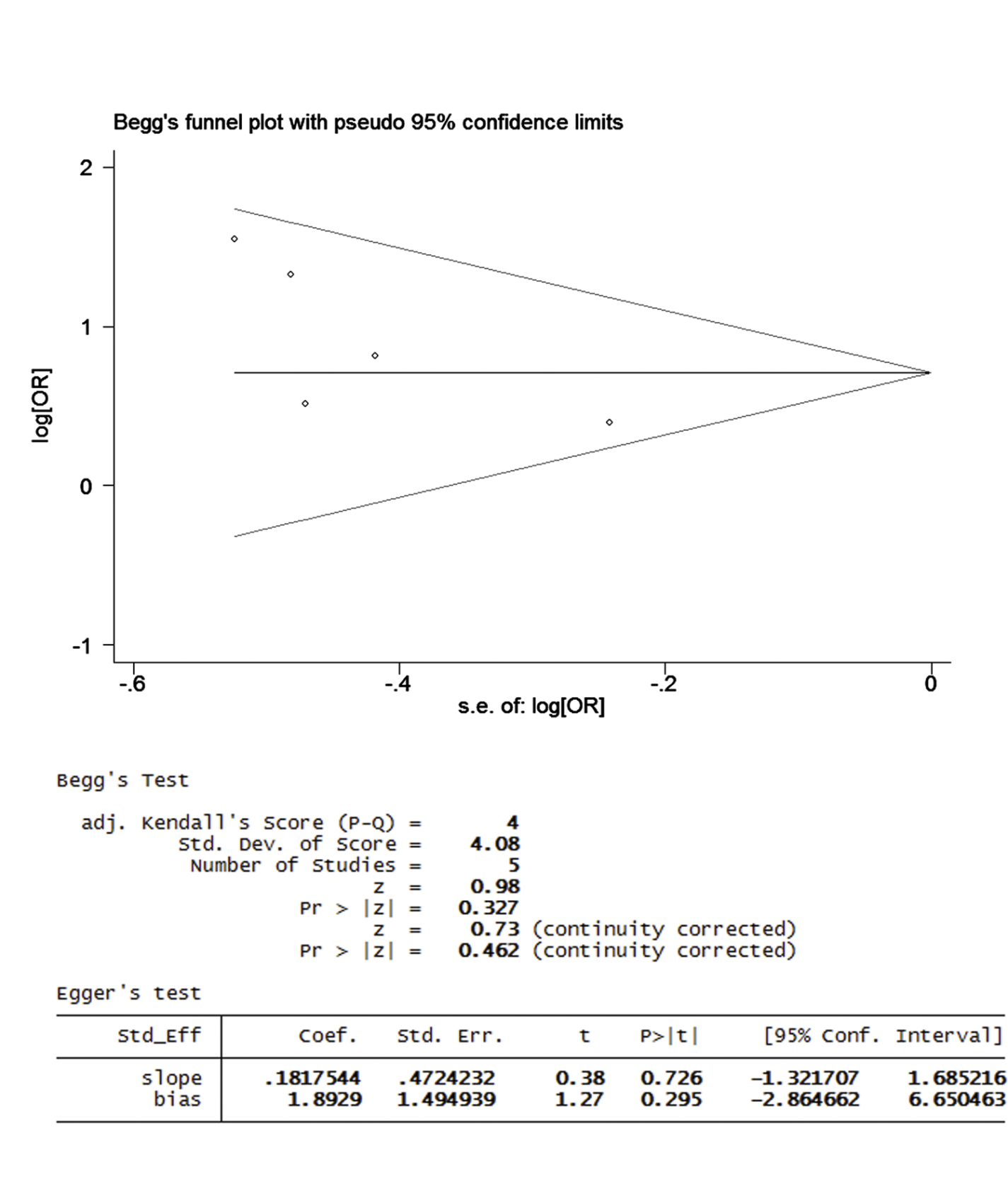

Supplement: Figure S4 — Begg’s funnel plot of publication bias for the discontinuation rate comparison with pseudo 95% confidence limits. Horizontal line represents the overall OR of the 5 RCTs. Spots represent each RCT. P = 0.295. No publication bias was identified. (TIF) [file pone.0052158.s004.tif]

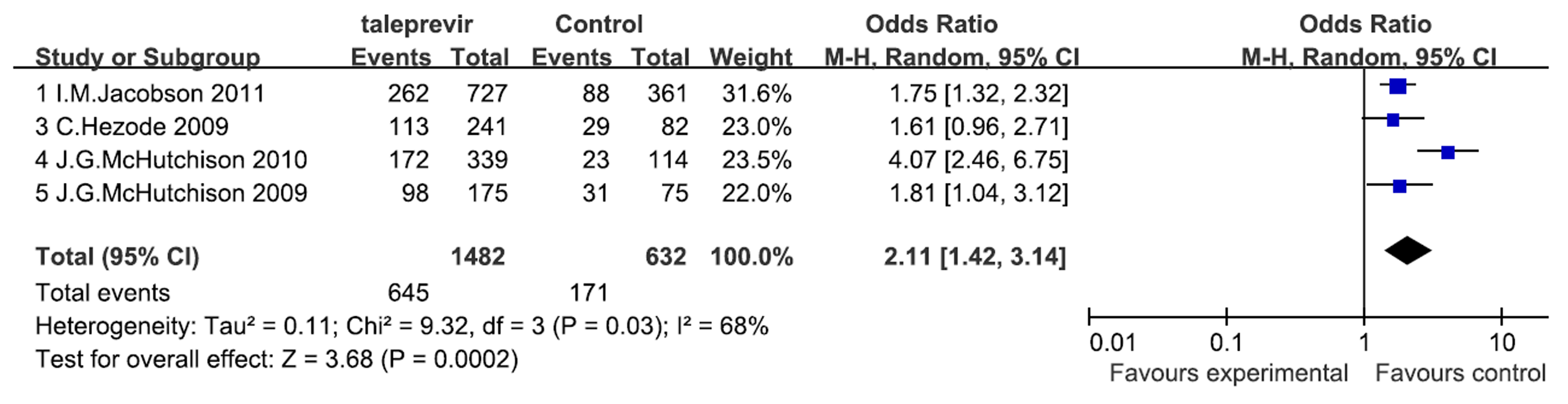

Supplement: Figure S5 — Meta-analysis of telaprevir plus peginterferon and ribavirin therapy on rash incidence. The incidence of rash was significantly higher in telaprevir group than in the PR group (OR = 2.11 [1.42, 3.14], P<0.001; I2 = 68%). Columns in green represent the mean difference of each study and column size represents the study weight in the analysis. Lanes represent the 95% CI of each study. Diamonds in black represent the overall effect size, and diamond width represents the overall 95% CI. (TIF) [file pone.0052158.s005.tif]

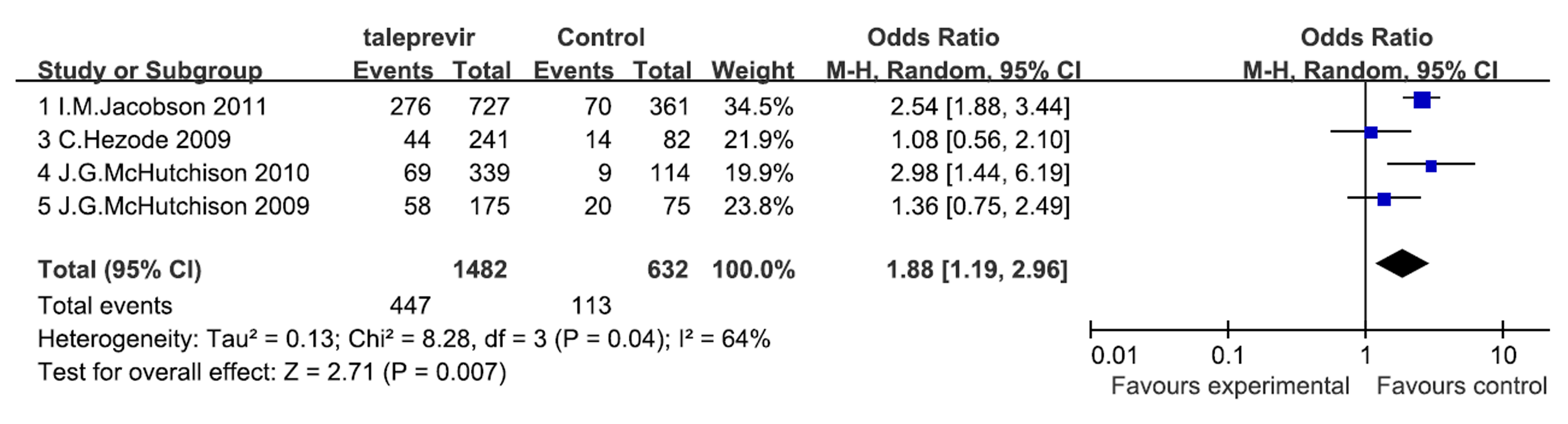

Supplement: Figure S6 — Meta-analysis of telaprevir plus peginterferon and ribavirin therapy on anemia incidence. The incidence of anemia was significantly higher in the telaprevir group than in the PR group (OR = 1.88 [1.19, 2.96], P = 0.007; I2 = 64%). Columns in green represent the mean difference of each study and column size represents the study weight in the analysis. Lanes represent the 95% CI of each study. Diamonds in black represent the overall effect size, and diamond width represents the overall 95% CI. (TIF) [file pone.0052158.s006.tif]

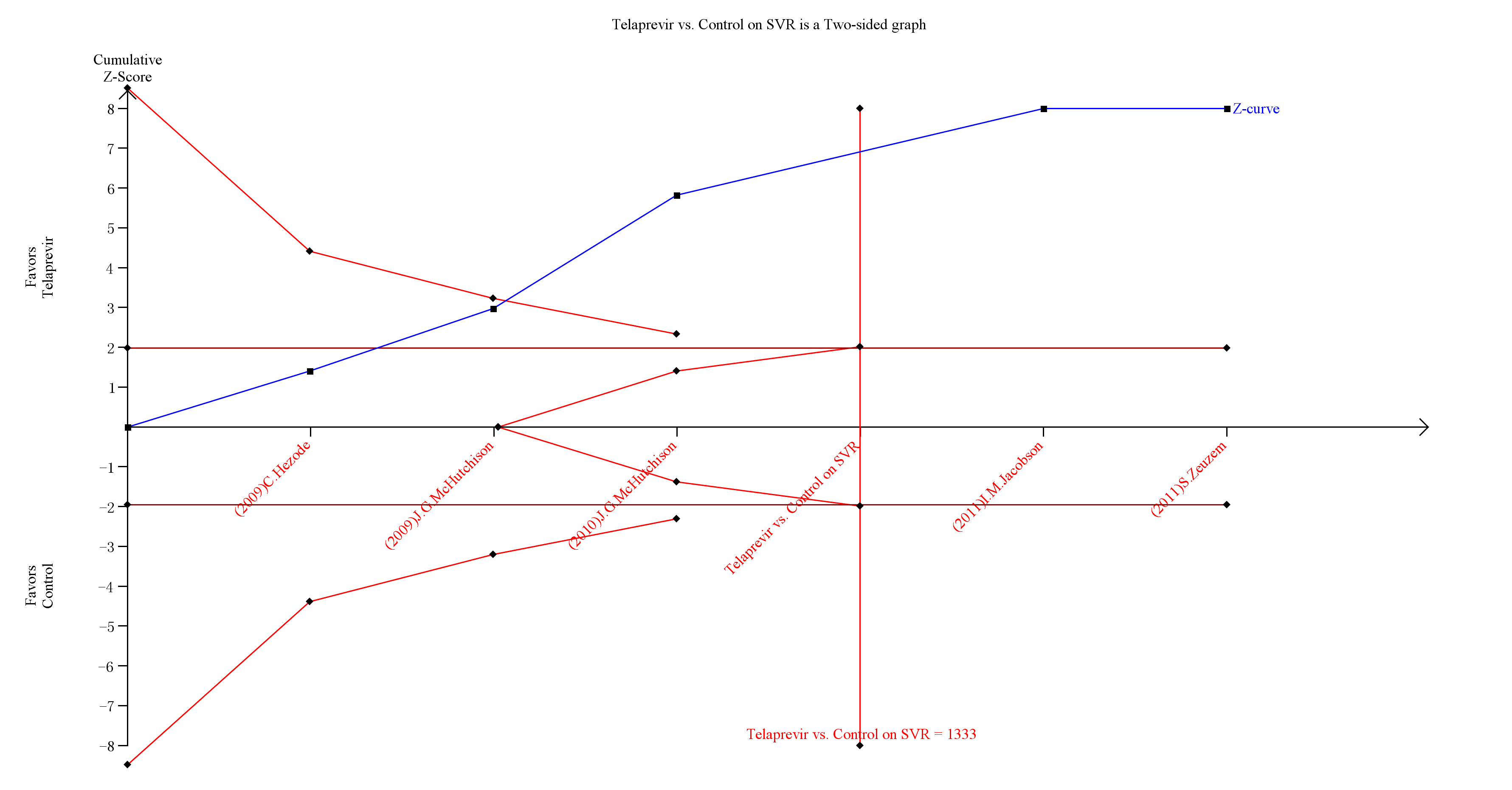

Supplement: Figure S7 — The heterogeneity-adjusted required information size to demonstrate or reject a 20% odds ratio reduction of telaprevir-based therapy (with a control group proportion of 32%, an alpha of 5%) is 1333 patients (vertical red line). The rede inward-sloping line to the left make up the trial sequential monitoring boundaries and the blue line is the cumulative Z-curve. (TIF) [file pone.0052158.s007.tif]
